# Soy Protein Isolate Improved the Properties of Fish Oil-Loaded Chitosan–Sodium Tripolyphosphate Capsules

**DOI:** 10.3390/foods14010086

**Published:** 2025-01-01

**Authors:** Yunning Wang, Mubeen Asad, Deqian Wang, Xiaofan Gao, Guoliang Zheng, Jian Zhong, Jing Xie, Zhengquan Wang

**Affiliations:** 1 Shanghai Engineering Research Center of Aquatic-Product Processing & Preservation, Laboratory of Quality & Safety Risk Assessment for Aquatic Products on Storage and Preservation (Shanghai), Ministry of Agriculture and Rural Affairs, College of Food Science and Technology, Shanghai Ocean University, Shanghai 201306, China; m220300952@st.shou.edu.cn (Y.W.); asadmubeen101@outlook.com (M.A.); m210300950@st.shou.edu.cn (D.W.); m230301006@st.shou.edu.cn (X.G.); m230351111@st.shou.edu.cn (G.Z.); jzhong@shsmu.edu.cn (J.Z.); jingx@shou.edu.cn (J.X.); 2 College of Biosystems Engineering and Food Science, Zhejiang University, 866 Yuhuantang Road, Hangzhou 310058, China; 3 Key Laboratory of on Site Processing Equipment for Agricultural Products, Ministry of Agriculture and Rural Affairs, Hangzhou 310058, China; 4 Xinhua Hospital, Shanghai Institute for Pediatric Research, Shanghai Key Laboratory of Pediatric Gastroenterology and Nutrition, Shanghai Jiao Tong University School of Medicine, Shanghai 200092, China

**Keywords:** electrospray, soy protein isolate, chitosan, microcapsules

## Abstract

In this paper, the effect of soybean isolate protein (SPI) content on the physicochemical properties and oxidative stability of chitosan–sodium tripolyphosphate (CS-STPP)-loaded fish oil capsules was investigated. The SPI/CS-STTP capsules formed after the addition of different amounts of SPI were larger in size and more homogeneous in morphology than the CS-STPP capsules, and the SPI was encapsulated on the surface of the CS matrix, altering the surface properties and morphology of the particles. The study of different CS-to-SPI blend ratios (1:0, 3:1, 2:1, 1:1, 1:2) showed that the water content of the microcapsules increased from 49.79% to 53.27–64.99%, the fish oil loading increased from 17.06% to 18.31–24.89%, and the encapsulation rate increased from 89.42% to 93.90–96.14%. In addition, the addition of SPI reduced the maximum peroxide value from 445 to 264 meq/kg oil. In the simulated in vitro digestion experiments, the addition of various amounts of SPI resulted in a significantly lower percentage of final free fatty acid (FFA) release than observed for CS-STPP capsules alone. These changes observed in the properties may be due to structural differences between CS-STPP capsules and SPI/CS-STPP capsules. All the results confirm that the obtained capsules are promising for the development of functional foods and drugs.

## 1. Introduction

In recent years, along with the explosion of human fishing resources and the processing and utilization of high-value aquatic products, which are by-products of fish processing, have become absolutely crucial directions in the field of food and nutrition [1]. At present, fish oil rich in n-3 polyunsaturated fatty acids such as eicosatetraenoic acid and docosahexaenoic acid is the most important high-value aquatic product, but since fish oil fatty acids contain multiple unsaturated double bonds that are highly susceptible to inactivation by oxidation and degradation, the difficulty of efficiently delivering these nutrients to the intestinal tract is a currently persistent problem in the application of fish oil [2,3]. Therefore, determining how to effectively deliver the nutrients in aquatic products to human digestive organs with high efficiency is a major challenge in the food industry.

Packaging technology is a very effective means of protecting and controlling the release of drugs, nutrients, and other active ingredients [4]. Common packaging technologies include lyophilization, spray drying, and electrospray technology [5,6,7]. Among them, electrospray technology, as a new, non-thermal processing and packaging technology, can package thermally unstable bioactive substances at room temperature and standard atmospheric pressure, such that fish oil/nutrients can enter the human intestine smoothly [8,9,10]. Our research team successfully prepared Ca^2+^–sodium alginate capsules and chitosan–sodium tripolyphosphate (CS-STPP)–Tween capsules coated with fish oil using the combined technology of electrospray–ionotropic gelation [11,12]. The electrospray–ionotropic gelation technique is a process that combines electric spraying with ionic gelation (ion-induced cross-linking) to form a gel-like structure [13].

CS is a biocompatible carrier and enables the stable encapsulation and controlled release of active ingredients [14]. It is the only naturally occurring positively charged polysaccharide and can complex with negatively charged proteins through electrostatic interactions, allowing for the preparation of microcapsules with CS as a carrier [15], such as probiotic bacteria encapsulated in electrospray alginate–CS and fish oil encapsulated in electrospray CS-STPP[(C_6_H_11_NO_4_)_n_·Na_5_P_3_O_10_ ]–Tween capsules [12,16]. Plant proteins are less allergenic and safer to use as wall materials than animal proteins for complex coagulation, which is essential to achieve the desired encapsulation efficiency and targeted release in the gut [17].

Soybean isolate protein (SPI) is one of the world’s most affordable and efficient natural plant proteins. It is the most popular commercially available plant protein ingredient that is both environmentally friendly and safe [18]. SPI/CS complexes have been shown to be effective in stabilizing emulsions and enhancing the stability of bioactive molecules through microencapsulation, thereby facilitating the in vitro delivery of nutrients, such as SPI/CS complex-encapsulated beef flavor (RBF) and microencapsulated garlic essential oil using SPI and CS as wall materials [19,20]. In addition, although our group has previously studied the effects of non-natural macromolecules, such as Tween and Genipin, on the microencapsulation of encapsulated fish oils [12,21], no research has been conducted on the microencapsulation of fish oils using SPI and CS as wall materials.

The aim of this study was to investigate the effect of the addition of natural SPI macromolecules on the properties of CS-STPP-loaded fish oil submillimeter capsules. First, we prepared SPI/CS-STTP and CS-STTP submillimeter capsules and investigated the effects of the SPI/CS composite ratio and voltage on fish oil-loaded capsules. Second, the effects of adding different levels of SPI on the physicochemical properties of CS-STTP capsules and their stability were investigated. Finally, the effects of adding different levels of SPI on the in vitro disintegration of CS-STTP in capsules were investigated to determine whether SPI/CS-STTP can be used for the encapsulation of thermally unstable substances in functional foods, improving in vitro transport efficiency and producing high-quality food products using soybean raw materials.

## 2. Materials and Methods

### 2.1. Reagents

Fish oil (food grade, DHA + EPA ≥ 70%) was obtained from Qianye Cao firms (Xian, China), and SPI (protein content > 90%, food grade) was provided by Shandong Shansong Biologicals (Linyi, China). CS (degree of deacetylation 90%, chemically pure grade) was provided by Shanghai Sinopharm Chemical Reagent (Shanghai, China) and STTP (analytical purity, 98% molecular weight 367.86) from McLean, Shanghai, China. Porcine pancreatic lipase was purchased from Shanghai Lantuo (Shanghai, China). Porcine bile extract and potassium iodide were purchased from Shanghai Macklin (Shanghai, China). Ethanol (AR grade, purity ≥ 95%, molecular weight 46.07) came from Sinopharm, Shanghai, China; acetic acid (AR grade, purity ≥ 99.5%, molecular weight 60.05) from Sinopharm, Shanghai, China; petroleum ether (25 mL, AR grade, 30–60 °C) from China National Pharmaceutical Group, Shanghai, China; and concentrated ammonia water (1.25 mL, AR grade) came from Sinopharm, Shanghai, China.

### 2.2. Preparation of SPI/CS Emulsion

Stable CS dispersions and SPI/CS emulsions were prepared by homogenization [22]. Three grams of CS was dissolved in 100 mL of 1% CH_3_COOH in water; another 3 g of SPI was dissolved in ultrapure water. The pH of the SPI solution was adjusted to 7.0 with 1 M HCl; the pH of the CS solution was adjusted to 5.2–5.4 with NaOH, and the SPI/CS was mixed at different ratios. The SPI/CS solution was then mixed at different ratios, and a quantitative amount of fish oil was added after mixing. The emulsion of the SPI/CS loaded with fish oil was obtained by homogenization at 8000 rpm for 60 s using an RCD-1A high-speed homogenizer. The droplet size of the emulsion was observed using an inverted optical microscope (Minz, Shanghai, China).

### 2.3. Preparation of Capsules

The SPI/CS-STPP stabilized capsules were prepared by the uniaxial electrospray-ion gel method using a custom-designed electrospray apparatus [23]. The ratios of the SPI/CS were 1:0, 3:1, 2:1, 1:1, and 1:2. The jet feeding rate was 30 µL/min. The internal diameter of the stainless needle used was 0.60 mm. The collection distance was 10 cm, different voltages (0 kV, 4 kV, 7 kV, 10 kV, and 21 kV) were applied, and the ambient temperature was 25 °C.

### 2.4. Observation of SPI/CS-STPP Capsules’ Morphology

The prepared capsules were transferred to a clean culture dish and photographed with a digital camera (DC). The photographs were then analyzed using ImageJ software (version 1.53c, National Institutes of Health, New York, NY, USA) to determine the size of these capsules. The capsule was dried for 1 h, and the platinum layer was sputtered for 2 min and observed using a scanning electron microscope (Hitachi S-3400 SEM, Tokyo, Japan) under a high vacuum with an acceleration voltage of 10 kV.

### 2.5. FTIR Analysis

FTIR experiments were carried out to investigate whether fish oil was embedded in the electrospray SPI/CS-STTP microcapsules. All investigations were carried out using the Spotlight 400 Fourier Transform Infrared Spectrometer (Perkinelmer Waltham, MA, USA) in the 4000–400 cm range with a resolution of 4 cm^−1^ [24].

### 2.6. Moisture Content

The freshly prepared microcapsules were washed in ultrapure water, removed after washing, and placed on clean aluminum foil. The moisture on the surface of the capsule was wiped off with absorbent paper and placed in a clean glass bottle at room temperature for 2 h. About 1 g of the capsule was removed and placed in a constant-weight bottle (m_1_), and the bottle with the capsule (m_2_) was measured, after which the sample was placed in an oven and repeatedly heated to a constant weight (m_3_) at 103 °C. Lastly, the moisture content of the capsule was calculated using the equation, and finally, the moisture content of the capsule using Equation (1) was calculated [12].

(1) $$Water\;content(\frac{\omega }{\omega },\%)=\frac{m_{2}-m_{3}}{m_{2}-m_{1}}\times 100$$

### 2.7. Loading Capacity and Encapsulation Efficiency

According to the Rozi Gottlieb method, freshly prepared SPI/CS-STPP capsules were placed on a clean glass plate, and surface water was absorbed with blotting paper; approximately 0.5 g (m_4_) was removed and placed in a liposuction bottle. After that, 1.25 mL of NH_3_H_2_O solution was added and heated in an SHJ-A4 water bath at a temperature of 60 °C for 5 min. A total of 10 mL of C_2_H_6_O was added to the mixture. After cooling, 25 mL of C_4_H_10_O and 25 mL of C_5_H_12_ were added sequentially. After cooling, 25 mL of C_4_H_10_O and 25 mL of C_5_H_12_ were added sequentially. The mixture was allowed to stand for 30 min, and the volume (V_1_) was read. Part of the ether layer (V_2_) was removed and placed in a flask of constant weight (m_5_), evaporated using a RE52AA rotary evaporator for 5 min, and then dried in a desiccator at 103 °C for 3 h. After cooling, the mixture was weighed repeatedly until a constant weight (m_6_) was obtained, and Equation (2) was used to calculate the loading capacity [25].

(2) $$LC(\%)=\frac{m_{6}-m_{5}}{m_{4}\times V_{2}/V_{1}}\times 100$$

After removing the surface moisture from the capsule, weigh 0.5 g (m_7_) and place it in a beaker. Rinse the beaker with 30 mL of light petroleum and filter through filter paper into a constant-weight rotary evaporator flask. Evaporate the solution completely using a rotary evaporator, place the rotary evaporator flask in an oven, and dry to a constant weight (m_8_). Use Equation (3) to calculate the encapsulation efficiency [26].

(3) $$EE(\%)=\frac{m_{7}LC-m_{8}}{m_{7}LC}\times 100$$

### 2.8. SPI/CS-STPP Capsules Storage Observation

The capsules were preserved by dry and wet methods. Dry preservation was conducted in a light-protected environment for 10 days, and wet preservation was conducted in a light-protected receptive solution for 10 days. The capsules were photographed with DC at different time points.

### 2.9. Oxidative Stability of Oil

We placed 0.5 g (m_9_) of the freshly prepared capsules in silica gel vials and placed them in a constant temperature and humidity (63 °C, 70% humidity) incubator to determine the PV. We took pictures of the capsules in the silica gel vials at 0, 3, 6, 12, 24, 48, and 72 h. The capsules in the vials were then rinsed with 30 mL of CH_3_COOH-C_8_H_18_ (3:2) and allowed to stand for 10 min after gentle shaking. The capsules in the vials were then rinsed with 30 mL of CH_3_COOH-C_8_H_18_ (3:2); the mixture was gently shaken and allowed to stand for 10 min, after which 1 mL of saturated KI solution was added, shaken for 40 s, and placed in a light-absorbing environment for 3 min. A hundred milliliters of ultrapure water was immediately added to the mixture and titrated with Na_2_S_2_O_3_ (0.001 mol/L). The titration was carried out simultaneously with the titration of Na_2_S_2_O_3_ (0.001 mol/L). The titration was carried out by stirring with a glass rod until the color became colorless, then 1 mL of starch solution (10 g/L) was added and stirred continuously until the blue color disappeared. A blank of the titration volume (V_4_) was used to calculate the PV according to the following equation. V_3_ is the volume titrated with Na_2_S_2_O_3_ and V_4_ is the volume consumed without the capsule and calculates the peroxide value from Equation (4) [27].

(4) $$PV(meq/\mathrm{kg}\;\mathrm{of}\;\mathrm{oil})=\frac{V_{3}-V_{4}}{m_{9}LC}\times 1000$$

### 2.10. Simulation of In Vitro Digestion

In model (1), 2 g of NaCl and 7 mL of HCl were dissolved in 1 L of water. After dissolving, 3.2 g of pepsin was added, and the pH was adjusted to 1.2 to prepare the simulated gastric fluid. Next, 1 g of microcapsules was removed and mixed with 15 mL of simulated gastric fluid to adjust the pH to 2.0. The mixture was shaken in a thermostatically controlled oscillating water bath at 100 rpm for 2 h at 37 °C, maintaining the pH at 2.0 (throughout the shaking process). Following this, the pH of the mixture was adjusted to 7.0 by shaking before the addition of porcine bile extract solution (3.5 mL, 54 mg/mL) and salt solution (1.5 mL, 10 mL CaCl_2_, 10 mL 150 mm NaCl). The pH was then adjusted to 7.0, after which 2.5 mL of the lipase solution (75 mg/mL) dissolved in phosphate buffer was taken and added to the previous mixed solution as a simulated small intestinal period (2 h). The pH was then adjusted every 30 min to maintain the pH at 7.0 and to record the volume of NaOH required to neutralize the FFAs released from lipid digestion.

In model (2), 1 g of dried capsule was added to 15 mL of ultrapure water, and the pH was adjusted to 7.0 in a water bath at 37 °C and 3.5 mL of bile extract (54 mg/mL). After that, 1.5 mL of salt solution (a mixture of 10 mm CaCl_2_ and 150 mm NaCl) was added, and the mixture was adjusted to a pH of 7.0. The capsule was dissolved in 2.5 mL of phosphate buffer (pH = 7). Then, 2.5 mL of freshly prepared lipase solution (75 mg/mL) dissolved in phosphate buffer (pH = 7) was added to the mixture. The pH of the mixture was monitored during 2 h of digestion in the small intestine, and the volume of NaOH (0.5 M) required to neutralize the FFA released from lipid digestion was recorded. Both model (1) and model (2) calculated the FFA using Equation (5) [28].

(5) $$FFA(\%)=100\times \frac{V_{NaOH}\times m_{NaOH}\times M_{fish\;oil}}{W_{Microcapsules}\times O_{c}\times 2}$$

### 2.11. Statistical Analysis

Three parallel experiments were performed to calculate the mean and standard deviation. One-way ANOVA was analyzed using SPSS version 17.0 (SPSS Inc., Chicago, IL, USA) and Origin 6.5 (MDI, Minneapolis, MI, USA) software.

## 3. Results

### 3.1. Preparation and Characterization of SPI/CS-STTP Capsules Loaded with Fish Oil

**Figure 1:**
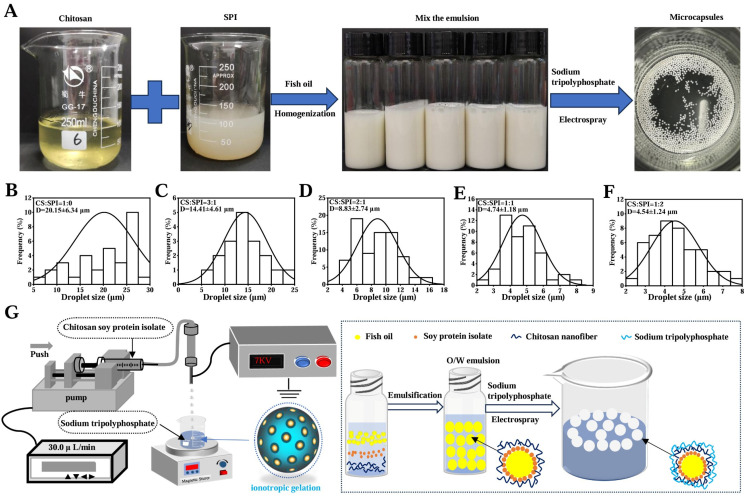
Preparation method and mechanism of SPI composite CS cross-linked STTP fish oil capsules. (**A**) Part of the experimental procedure for encapsulating fish oil with SPI/CS-STTP. (**B**–**F**) Particle size distribution frequency diagram of SPI/CS/fish oil lotion droplets prepared with different proportions of CS and SPI; the proportions are 1:0, 3:1, 2:1, 1:1, and 1:2, respectively. (**G**) Schematic diagram and formation mechanism diagram of the electric spray capsule preparation process.

First, we coalesced the fish oil-containing SPI and CS complexes to form an emulsion. Then, we cross-linked them with STTP using electrospray technology and deionized gel technology to produce fish oil-embedded SPI/CS-STTP capsules, which typically form stable beige dispersions, as shown in Figure 1. The emulsification phenomenon due to the ‘droplet interfacial tension decrease’ was clearly seen at 12 h [29]. At 72 h, a demarcation line became clearly visible in the dispersions a, and the droplet size significantly decreased under the optical microscope. As a result, the mixture needed to make SPI/CS-STTP capsules that were freshly prepared, as illustrated in Figure 1B–F. The emulsification degree increased over time. After the SPI/CS composite was formed, the particle size of the emulsion decreased as the SPI content increased. This is likely because more protein molecules adsorbed onto the droplet interface, forming a denser interfacial film. It decreased the droplet particle size by preventing droplet aggregation as its strength and elasticity increased [30].

**Figure 2:**
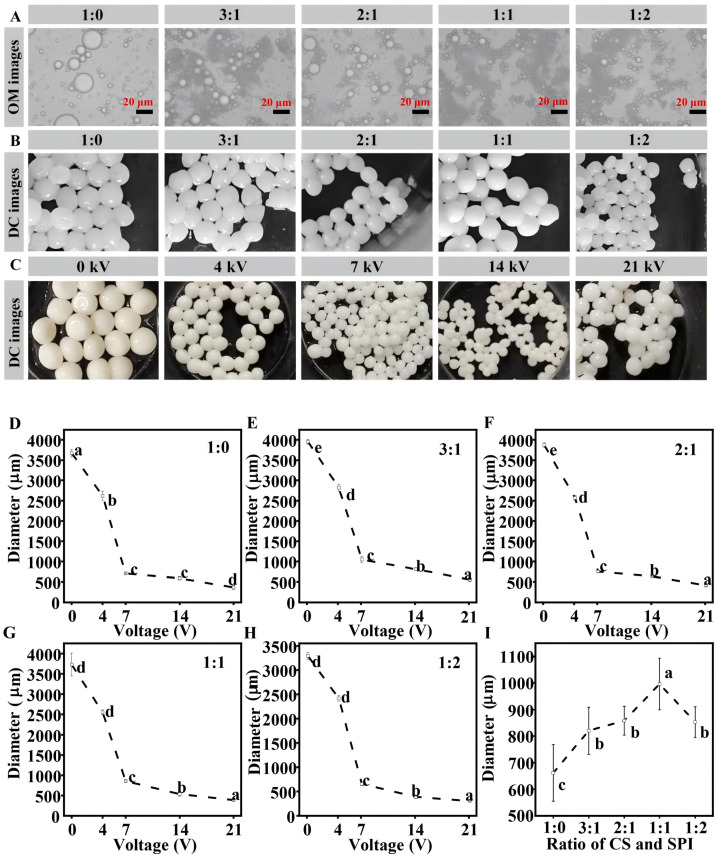
Investigation of the effects of adding different amounts of SPI and applying different voltages on the size of prepared fish oil capsules. (**A**) The optical microscope (OM) image of the lotion prepared from CS and SPI in different proportions was used to observe the appearance. (**B**) DC images of fish oil capsules prepared with different blend ratios of CS and SPI. (**C**) Digital camera images of fish oil capsules prepared by applying different voltages under the condition of 2:1 composite ratio of CS and SPI. (**D**–**H**) Size chart of capsules prepared at different voltages and different composite ratios. (**I**): Size chart of microcapsules prepared with different composite ratios of CS and SPI at a voltage of 7 kV. Different letters in Figure 2 indicate significant differences (*p* < 0.05).

Additionally, because the SPI also has surface activity, it can significantly reduce the oil–water interfacial tension, and this effect intensifies as its content increases. The interfacial tension is further reduced, which contributes to the further dispersion of droplets in the emulsification process and the formation of smaller droplets. However, because protein molecules aggregate with one another and the interfacial adsorption of droplets becomes saturated, if the SPI content is too high, the droplet size may not decrease anymore or may even increase. Additionally, more SPI enters the aqueous phase and alters the emulsion’s physical characteristics [31]. The SPI/CS-STTP capsules encapsulated with fish oil can be clearly seen in the DC image, which is uniform in size and has a rounded appearance. Additionally, transparent bright spots can be clearly seen on the spheres, similar to the results of the previously studied CS-STTP capsules loaded with fish oil [12]. As a result, the SPI/CS-STTP capsule is also a multinucleated, sub-millimeter-sized capsule. As shown in Figure 2A,B, all CS dispersions and SPI/CS emulsions consisted of microscopic droplets. Since the droplets of the CS dispersions were much larger than those of the SPI/CS emulsions, the addition of SPI increased the ability of the CS-STTP capsules to encapsulate the fish oils and reduced the droplet size, as shown in Figure 2C–H. The effect of applying different voltages on the appearance and size of the SPI/CS-STTP capsules was studied at a CS and SPI complex ratio of 2:1. The capsule size decreased exponentially with increasing applied voltage. This trend was similar to that of alginate-Ca^2+^ capsules and CS-STTP capsules loaded with fish oil via electrospray [11,12]. At a higher electrospray voltage, the capsules formed were smaller, likely due to the stronger electric field. The droplets were subjected to increased electrostatic repulsive force and electrodynamic force. The droplets at the nozzle were fragmented into smaller particles under the electrostatic field [32]. The effect of different volume ratios of SPI/CS dispersions on the appearance and size of SPI/CS-STTP capsules loaded with fish oil at an applied voltage of 7 kV was then investigated. It was found that the size of the microcapsules became larger with increasing SPI content, with the maximum size at an SPI/CS ratio of 1:1, and then decreased, as shown in Figure 2I. Since the intrinsic properties of the feed polymer solution and instrumental parameters are the main factors affecting the particle size, in this study, with the external factors held constant, the SPI/CS volume ratio was the sole variable affecting capsule size, which was linked to the polymer’s molecular weight [33].

### 3.2. ATR-FTIR Analysis of Electrospray Microcapsules

**Figure 3:**
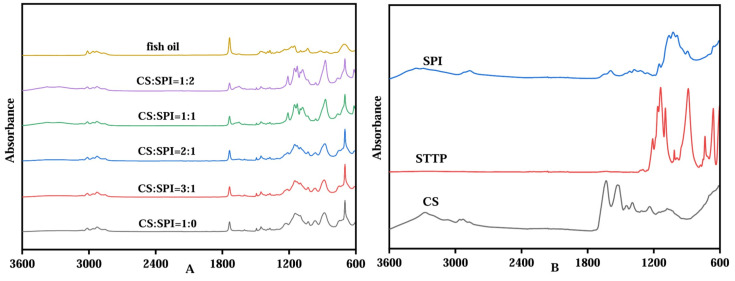
ATR-FTIR of SPI/CS-STTP capsules: (**A**) ATR-FTIR of fish oil and SPI/CS-STTP and CS-STTP capsules loaded with fish oil; (**B**) ATR-FTIR of CS, SPI, and STTP.

FTIR spectroscopy can be used to verify whether nutrients, such as fish oil, are successfully encapsulated [24]. As shown in Figure 3A, the FTIR spectrum of fish oil shows a peak at 3013.3 cm^−1^, with characteristic bands at 2964.4 cm^−1^ and 2932.1 cm^−1^. The FTIR spectra of the fish oil previously studied typically showed absorption peaks of the C-H stretching vibration at 3000 cm^−1^, indicating the presence of unsaturated fatty acids [34]. In addition, a particularly prominent peak was observed at 1735.1 cm^−1^, indicating the carbonyl (C=O) extension of the ester functional groups derived from fatty acids and lipids. The FTIR spectrum of the SPI/CS-TTP capsules loaded with fish oil shows fluctuations in the range of 3047.4–2950.6 cm^−1^, which are lower than the peak intensity of fish oil—the higher peak at 2926.3 cm^−1^ results from the presence of SPI and CS [35]. The peak intensity in the 1735.1 cm^−1^ region of the SPI/CS-STTP and CS-STTP capsules is significantly lower than that of fish oil. This indicates that the addition of SPI, CS, and STTP reduces the C=O vibration, confirming that fish oil has been encapsulated in the composite material, thereby reducing its direct contact with infrared light. New characteristic peaks in the range of 1210.3–874.5 cm^−1^ were observed for the SPI/CS-STTP and CS-STTP capsules, demonstrating the complexation between CS, SPI, and STTP during the encapsulation process. In conclusion, it has been demonstrated that fish oil can be successfully encapsulated in SPI/CS-STTP capsules [36].

### 3.3. Physicochemical Characterization of SPI/CS Capsules

**Figure 4:**
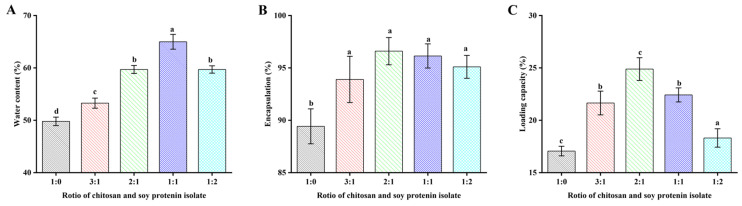
Physical and chemical properties of SPI/CS capsules containing fish oil: (**A**) Percentage of moisture content; (**B**) fish oil encapsulation efficiency; (**C**) fish oil loading capacity. There were significant differences (*p* < 0.05) in the use of different letters.

The moisture content of the SPI/CS-STTP capsules was determined by drying the samples at 103 °C to a constant weight [37]. Capsules prepared with different CS and SPI ratios (1:0, 3:1, 2:1, 1:1, and 1:2) turned yellow and eventually brown due to water evaporation, but at different rates, enabling the determination of water content, as shown in Figure 4A. The water content of the capsules depended on the CS and SPI composite ratio, increasing with higher soy isolate protein content and reaching the highest value (64.99 ± 1. 41%) at an SPI/CS ratio of 1:1, where the capsule size also reached its maximum. This was attributed to the greater water-holding capacity of SPI compared to CS, especially in capsules with a high oil loading capacity [38]. The fish oil acted as a water vapor transmission barrier, reducing water evaporation, increasing the hydrophobicity of the capsules, and limiting the transfer of water molecules [39].

In addition, the loading capacity (LC) and encapsulation efficiency (EE) are key parameters for assessing the encapsulation ability of fish oil in microcapsules. As shown in Figure 4B, the LC values increased and then decreased with increasing soybean isolate protein content. CS:SPI = 1:0 < 1:2 < 3:1 < 1:1 < 2:1 (17.06 ± 0.46% < 18.31 ± 0.87% < 21.65 ± 1.13% < 22.42 ± 0.67% < 24.89 ± 1. 08%), and the maximum LC value (24.89 ± 1.08%) was found at a CS:SPI ratio of 2:1, which was higher than that at a CS:SPI ratio of 1:0 (17.06 ± 0.46%), indicating that the SPI could significantly increase the fish oil loading rate of CS-STPP capsules. As CS and SPI form a complex through electrostatic interaction, the strength and toughness of the interfacial membrane of the microcapsule improve. This improved interfacial membrane can more effectively encapsulate the oil phase and reduce the leakage of oil droplets. Additionally, the added SPI can further participate in the cross-linking reaction between CS and STTP through its functional groups (e.g., carboxyl groups or amines), increasing the density and stability of the cross-linking network once the capsules are denser, forming a more stable three-dimensional network structure, which helps to maintain the shape and stability of the microcapsule. The microencapsulated structure is denser and forms a more stable three-dimensional network structure, which helps to maintain the shape and prevent leakage of fish oil [29,40]. As Figure 4C shows, the proportion of SPI increases and the EE values also increase, with all EE values exceeding 85%: CS:SPI = 1:0, 3:1, 2:1, 1:1, and 1:2 (89.42 ± 1.67%, 93.90 ± 2.20%, 96.60 ± 1.30%, 96.14% ± 1.15%, and 95.10 ± 1.09%). The reason for the difference in EE values depends specifically on the amount of SPI added, which is similar to the results for coffee oil encapsulated with sodium alginate-soya bean isolate [38]. In simple terms, EE largely depends on the composition and stability of the feed emulsification, which is highest when the CS:SPI ratio is 2:1.

### 3.4. Structural Analysis of SPI/CS-STTP Capsules

**Figure 5:**
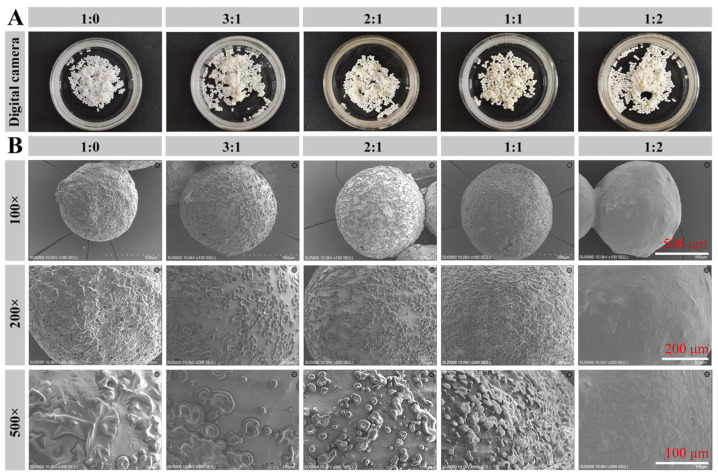
Appearance and structure information of fish oil SPI/CS capsules: (**A**) Digital camera images of oil-loaded microcapsules prepared by different composite ratios of CS and SPI; (**B**) SEM images of SPI/CS-STTP capsules prepared at different blend ratios of chitosan and soy protein isolate.

Figure 5A shows a newly prepared SPI/CS-STTP capsule, and its surface was examined using SEM. As shown in Figure 5B, wrinkle-like protrusions appeared on the surface of all CS-STTP and SPI/CS-STTP capsules, except for those with a CS:SPI ratio of 1:2, which resembled the results of the CS-STTP capsules [12]. As the SPI content increased, the surface protrusions became more numerous, like the fish oil capsules with ribose cross-linked SPI. These structural features indicate a protein matrix and the formation of multinucleated microcapsules. The surface of the microcapsules with a CS:SPI ratio of 2:1 was relatively smooth but slightly distorted, possibly due to more tightly aggregated proteins, which led to the formation of larger protrusions. [41]. Based on the previous studies and the above conclusions, we can, therefore, introduce the formation mechanism of the SPI/CS-STTP capsules. The water in the SPI/CS/fish oil droplets evaporates in the air as they are released from the needle by an electrospray machine. Afterward, a capsule filled with fish oil is produced when the SPI/CS contracts and fuses with a few fish oil droplets [42]. Finally, when the capsule droplets are in the STTP solution, ionotropic gelation occurs between CS and STTP, forming iono-gel-based CS-STTP or SPI/CS-STTP capsules. These capsules are more homogeneous than CS-STTP capsules and have higher oil loading and encapsulation rates.

### 3.5. Stability of SPI/CS-STTP Capsules

**Figure 6:**
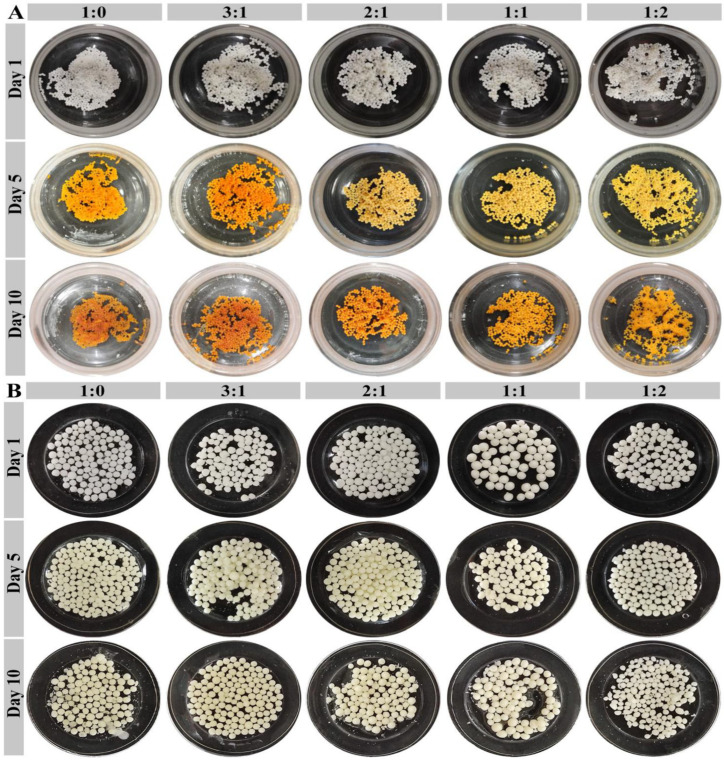
Stability of SPI/CS-STTP capsules loaded with fish oil in the dark at room temperature: (**A**) Under environmental conditions; (**B**) when stored in STTP receiving solution, transfer the capsule to a 35 mm culture dish with a 15 mm central opening and photograph—use of fish oil-loaded SPI/CS-STTP capsules as a control.

The morphological stability of the capsules is not only related to the quality of fish oil storage but also prevents leakage of fish oil during transport and storage in the human body, which plays a crucial role in their potential application in the food and pharmaceutical industries [25]. In this study, the CS-STTP and SPI/CS-STTP capsules were stored under ambient conditions and in STTP solution, respectively, and then photographed. As shown in Figure 6A, under ambient conditions, the SPI/CS-STTP capsules shrank by day 5, and their color changed to yellow. This could be due to dehydration of the SPI/CS phase and leakage of fish oil. In contrast, after ten days of storage, the capsules in 10% STTP solution only developed a pale-yellow surface, with the result seen in Figure 6B. Thus, the stability of both SPI/CS-STTP capsules in the receiving solution was better than that of the capsules under ambient conditions, which is similar to the storage effect of CS-STTP loaded with fish oil [12].

**Figure 7:**
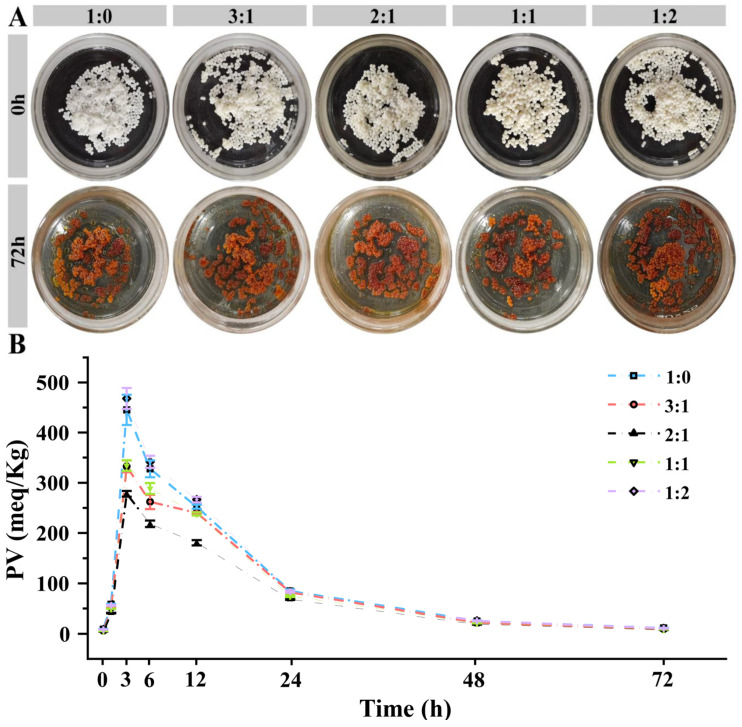
PV Schematic diagram of SPI/CS-STTP-loaded fish oil capsules prepared by different blend ratios of CS and SPI (63 °C, 72 h): (**A**) DC images; (**B**) PVs.

Oxidation of fish oil is a common problem during the storage of loaded fish oil capsules. Determining lipid PVs by testing in a constant temperature and humidity incubator at 63 °C is a common method for studying the oxidative stability of oils. One day of storage at 63 °C is comparable to sixteen days of storage at ambient temperature [28]. The color of the capsules changed from beige to brown, similar to that of the CS-STTP capsules [12]. The PVs of the SPI/CS-STTP capsules increased exponentially within 3 h, then decreased with time, which is consistent with our previous studies on the PV trends in calcium alginate capsules containing fish oil, CS-STTP, and gelatin anionic polysaccharide [11,12,21]. The change in color and PV is due to the formation of primary lipid hydroperoxides, followed by oxidation into secondary products [43]. Previous work has shown that Tween improves the stability of fish oil-loaded capsules, and the type of polymer affects the PV [12]. Therefore, studying the effect of the appropriate SPI/CS ratio on the oxidative stability of fish oil capsules may help in the development and application of loaded fish oil microcapsules. The oxidative stability of the oil was analyzed by measuring the PVs of SPI/CS-STTP capsules and CS-STTP capsules at 63 °C. As shown in Figure 7A, the color of the capsule changed from yellow to brown, and the PVs of the SPI/CS-STTP capsules followed a similar trend to those of the CS-STTP capsules over time. As shown in Figure 7B, the PVs after 3 h were lower than those of the CS-STTP capsules after 3 h. Therefore, the addition of SPI improves the oxidative stability. The PVs for different ratios of SPI/CS were as follows: CS:SPI ratio 1:2 > 1:0 > 1:1 > 3:1 > 2:1 (468 > 445 > 334 > 322 > 284 meq/kg oil). The SPI is able to trap the intermediates generated during lipid oxidation and reduce the further reaction of these species with their fish oils. SPI can also interrupt the free radical chain reaction of lipid oxidation, thereby significantly reducing the accumulation of peroxides. However, excessive amounts of SPI may not be able to fully participate in the complex, resulting in a loose or uneven membrane structure, but rather increase the permeability of oxygen, thus accelerating the oxidation reaction and increasing the peroxide level [44]. Therefore, the oxidative stability of the capsules is best when the composite ratio of CS and SPI is 2:1.

### 3.6. Effect of Different Ratios of SPI/CS on In Vitro Digestive Behavior

**Figure 8:**
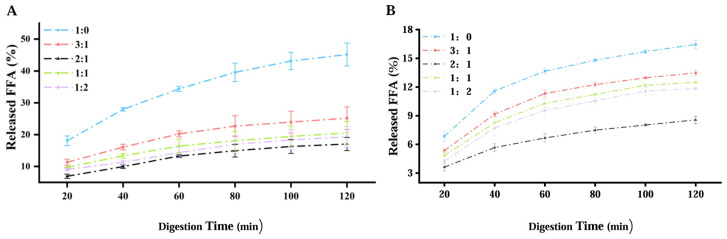
Percentage of FFA released from SPI/CS-STTP-loaded fish oil capsules in different proportions during the small intestine phase of gastrointestinal models: (**A**) Gastrointestinal model; (**B**) small intestine model.

Digestion of the resulting capsules in the human digestive system mainly involves the mouth, stomach, and small intestine. The millimeter-sized capsules remain in the mouth for only a few seconds when swallowed with water, making the oral digestive behavior of the capsules negligible [11]. The present study focused on the in vitro digestibility of the capsules in the gastrointestinal tract model and the small intestine model. At the end of the experiment, the spherical structure of all capsules remained undamaged, consistent with calcium alginate/Span capsules [25]. In the small intestine phase of the gastrointestinal model, the order of the percentage of FFA released from the capsules was CS:SPI = 1:0 > 1:2 > 1:1 > 3:1 > 2:1. As shown in Figure 8A,B, after adding SPI, the percentage of the FFA finally released from the SPI/CS-STTP capsules was significantly lower than that from the CS-STTP capsules in both the gastrointestinal tract and small intestine models. This suggests that the SPI improved the in vitro digestive stability and retention time, enhancing the bioavailability of fish oils, similar to the results observed in the SPI/CS encapsulation of rutin [45]. The combination of SPI and CS improved capsule stability, preventing aggregation and hydrolysis of fat droplets [46]. Additionally, SPI enhanced the physical protective layer of the capsules, slowing the action of digestive enzymes and reducing the release, thereby reducing FFA accumulation during digestion [47]. Excessive SPI can lead to excessive swelling or rupture of the capsule’s membrane, as the membrane structure is not dense enough, and the oil phase is more likely to be released during in vitro digestion [48]. The FFA percentage was the lowest when the CS/SPI ratio was 2:1, indicating that the optimal ratio of CS to SPI is 2:1.

## 4. Conclusions

In this study, SPI/CS-STTP capsules loaded with fish oil were successfully produced for the first time using electric spraying and composite coagulation technology. The impact of the SPI addition on the performance of fish oil-loaded CS-STTP capsules was thoroughly investigated. Compared to CS-STTP/Tween capsules, the SPI/CS-STTP capsules exhibited a more uniform and rounded appearance, along with a significantly higher oil loading rate. The oil loading rate reached 24.89 ± 1.08% at a CS/SPI volume ratio of 2:1. The incorporation of SPI not only enhanced the antioxidant capacity of the capsules but also improved their stability and controlled oil release in simulated gastrointestinal digestion environments. Additionally, microstructural analysis revealed the formation of a more compact and uniform biopolymer network with the inclusion of SPI. However, there are several aspects of future research that deserve further investigation. Firstly, there is a need to evaluate the long-term stability of SPI, the impact of different SPI and CS-STTP ratios on packaging scalability and product economics, and the biodegradability of SPI/CS-STTP and its environmental impact. By addressing these research gaps, the potential of SPI/CS-STTP capsules as functional food ingredients can be fully realized.

## Data Availability

The original contributions presented in this study are included in the article. Further inquiries can be directed to the corresponding author.
